# Obituary

**Published:** 1904-06-15

**Authors:** 


### OBITUARY.

### DR. EDGAR DENMAN SWAIN.

Colonel Edgar D. Swain died Thursday afternoon, April 28, 1904, as the result of a stroke of paralysis from which he suffered in the morning.

His death was a shock to his friends, who thought him to be enjoying the best of health, but it is believed the exertion of moving to another residence taxed the Colonel's strength and brought on the attack of paralysis while dressing Thursday morning at 6 o'clock. Restoratives were of no avail and he lapsed into unconsciousness, passing peacefully away about 2 o'clock in the afternoon.

Edgar D. Swain was born August 14, 1836, in Westford, Vermont, and came to Illinois in 1859.

We quote from the *Review*:

In the profession of dentistry his usefulness and his honors have been conspicuous. Soon after coming to Chicago to practice (after the close of the war), he identified himself with the group of faithful men who maintained the Chicago Dental Society and the Illinois State Dental Society for thirty years or more, and he filled at various times every position of responsibility or of honor which either of them had to bestow, and his name was scarcely ever absent from their lists of officers or committees. At the time of his death he was chairman of the committee on infractions of the code of ethics of the State Society. He received many years ago the honorary degree of D. D. S. from the Ohio College of Dental Surgery. He helped to organize the Chicago Dental Infirmary which soon became the Chicago College of Dental Surgery, and served for some years as its secretary and treasurer. He was one of the group of men who in 1891 organized the present North-western University Dental School, and was seven years dean of its faculty.

Some years ago severe and increasingly frequent attacks of rheumatism forced him to retire from practice and he went to Seneca Falls, N. Y., and engaged in out-of-door employment, raising poultry, fruits, etc. This enterprise had to be given up because his wife became a helpless invalid from the effects of a stroke of paralysis, and they moved to Batavia, Illinois. During the period of more active out-of-door life, Dr. Swain entirely recovered and appeared to regard himself, and was thought by his friends to be in excellent health. He had resumed the practice of dentistry in Batavia. His sudden death has therefore come with a great shock of surprise and grief to his host of friends. His place in the affections of his comrades and professional associates can never be filled.—*American Journal.*

### MEMORIAL TO DR. TAFT.

THE American Dental Society of Europe at its last meeting adopted the following expression of its sentiments on the death of Dr. Taft.

WHEREAS, There departed on the 15th of October, 1903, a life well rounded out in good works, in the death of Dr. Jonathan Taft, of Ann Arbor, Michigan, at the age of 83 years, it is hereby

RESOLVED, That in his death, the American Dental Society of Europe has occasion to mourn the loss of its most illustrious member. He was one of those who held a unique position, linked between ignominy and vigorous manhood in the history of our profession.

RESOLVED, That in his passing away from this sphere of usefulness, the oldest editor and teacher in the history of our calling has paid the debt of nature.

RESOLVED, That Mrs. Taft be assured of the sincere sympathy this Society feels for her in her sad bereavement, and the Secretary is instructed to forward a copy of these resolutions to her, and the same shall be engrossed with other minutes and published as a part of the transactions of this meeting.

(Signed) N. S. JENKINS,
G. C. DABOLL,
WM. MITCHELL, *Committee.*

